# The emerging role of TET enzymes in the immune microenvironment at the maternal-fetal interface during decidualization and early pregnancy

**DOI:** 10.3389/fimmu.2022.1066599

**Published:** 2023-01-05

**Authors:** Mengmeng Jin, Jianxiong Ji, Xi Chen, Ying Zhou, Dimin Wang, Aixia Liu

**Affiliations:** ^1^ Department of Reproductive Endocrinology, Women’s Hospital, Zhejiang University School of Medicine, Hangzhou, China; ^2^ Key Laboratory of Reproductive Genetics (Ministry of Education), Zhejiang University, Hangzhou, China; ^3^ Department of Neurosurgery, The Second Affiliated Hospital of Zhejiang University School of Medicine, Hangzhou, Zhejiang, China; ^4^ Department of Reproductive Medicine, People's Hospital of Xinjiang Uygur Autonomous Region, Urumqi, Xinjiang, China

**Keywords:** DNA methylation, TET enzymes, decidualization, early pregnancy, microenvironment, immune cells

## Abstract

A dysregulated immune microenvironment at the maternal-fetal interface in early pregnancy may lead to early pregnancy loss, fetal growth restriction, and preeclampsia. However, major questions about how epigenetic modifications regulate the immune microenvironment during the decidualization process and embryo implantation remain unanswered. DNA methylation, the main epigenetic mechanism involved in the endometrial cycle, is crucial for specific transcriptional networks associated with endometrial stromal cell (ESC) proliferation, hormone response, decidualization, and embryo implantation. Ten-eleven translocation (TET) enzymes, responsible for catalyzing the conversion of 5-methylcytosine to 5-hydroxymethylcyosine, 5-formylytosine, and 5-carboxylcyosine to achieve the DNA demethylation process, appear to play a critical role in decidualization and embryo implantation. Here, we provide a comprehensive view of their structural similarities and the common mechanism of regulation in the microenvironment at the maternal-fetal interface during decidualization and early pregnancy. We also discuss their physiological role in the decidual immune microenvironment. Finally, we propose a key hypothesis regarding TET enzymes at the maternal-fetal interface between decidual immune cells and ESCs. Future work is needed to elucidate their functional role and examine therapeutic strategies targeting these enzymes in pregnancy-related disease preclinical models, which would be of great value for future implications in disease diagnosis or treatment.

## Introduction

The endometrium, regarded as one of the most dynamic tissues in the human body, undergoes periodic changes, including cell proliferation, differentiation, and apoptosis (1). This tissue is composed of luminal and glandular epithelial cells, stromal cells, immune cells, endothelial cells, and so on, participating in the formation of the microenvironment at the maternal-fetal interface and controlling the subsequent invasion of trophoblast cells and the establishment of the maternal-fetal interface immune tolerance (2, 3). Abnormal decidualization of the endometrium can lead to infertility and a variety of pregnancy-related diseases, including early pregnancy loss (EPL), fetal growth restriction (FGR), and preeclampsia (PE) (4, 5). At present, studies have shown that a variety of steroid hormones, transcription factors, lipids and cell cycle–related proteins regulate the process of decidualization and participate in early embryo implantation and pregnancy maintenance. Epigenetic modification is an important regulation mode that affects gene expression and cell function. It can occur at transcriptional, posttranscriptional, and posttranslational levels, involving DNA methylation, histone methylation, and histone acetylation and deacetylation. At present, there is a large amount of evidence that epigenetic modification is involved in the regulation of the decidualization process (6).

DNA methylation is one kind of chemical modification of DNA, by which the cytosine nucleotide is converted into 5mC by a family of DNA methyltransferases (7). Whereas DNA demethylation is generated by active enzymatic demethylation during which 5-methylcytosine (5mC) undergoes a series of oxidation reactions catalyzed by the methylcytosine dioxygenases ten-eleven translocation (TET) enzymes (8). High levels of CpG islands and methylation of these islands may result in transcriptional silencing or repressing (7, 9, 10). Thus, DNA methylation is considered to be a main mechanism behind many fundamental cellular processes, including the endometrium’s cyclical changes (11). The tissue-specific variation in DNA methylation content across the menstrual cycle further suggests that DNA methylation regulates gene expression during the endometrial cycle (7, 12, 13).

Of note, the creation of an appropriate immune microenvironment is another key element for blastocyst implantation (14, 15). The decidual immune cells are mainly composed of lymphocytes (e.g., NK cells, T cells, dendritic cell and NK-T cells, etc.) and macrophages, and their proportion changes in the endometrial cycle (16). Dysregulation of the immune response and immune cell distribution may lead to placentation failure and reproductive decline (17, 18). Studies have shown that the genes affected by decreased methylation during decidualization were mainly associated with immune response regulation (19, 20). Furthermore, DNA methylation also plays a vital role in immune cell development and maturation, which contributes to decidual immune homeostasis (21–23). Altogether, we performed a comprehensive literature review concerning the roles of TET enzymes in the microenvironment at the maternal-fetal interface during decidualization and early pregnancy.

## Common features of TET proteins

TET1, TET2, and TET3, which constitute a family of iron (II)/2-oxoglutarate-dependent dioxygenase, are responsible for catalyzing the conversion of 5mC to 5-hydroxymethylcyosine (5hmC), 5-formylytosine (5fC), and 5-carboxylcyosine (5caC) to achieve the DNA demethylation process (24–26). These enzymes are associated with several conserved signaling pathways in several kinds of organs or tissues during development, especially in embryo and cancer development (27, 28).

### Structural similarities

TETs are all composed of an acatalytic region in their C-terminal that is responsible for 5mC dioxygenase activity termed the double-stranded β-helix domain and a conserved cysteine-rich domain, which is thought to be essential for proper folding. However, TET1 and TET3 carry a CXXC domain at the N-terminal region, which is not present in TET2. As a result, CXXC was separated and originates the IDAX gene, which acts as a negative regulator for TET2 (29).

### Functions in DNA demethylation and decidualization and early pregnancy

During the DNA demethylation process, TET enzymes oxidize the methyl group to 5hmC, 5-formylcytosineand 5-carboxylcytosine (30–33). After being recognized and excised by the enzyme thymine DNA glycosylase, these bases are substituted by an unmodified cytosine by base excision repair and lose their indicated function (33). In other words, TET enzymes work as erasers in the DNA methylation machinery during the whole endometrial cycle (34). All three TET enzymes are detectable in both epithelium and stroma tissues during the cycle. Besides this, recent studies show that TET1 and TET3 are preferentially expressed in the midsecretory phase over the other phases (27). Moreover, progesterone induces expression levels of all TET enzymes in endometrial epithelial cells, whereas estradiol plus progesterone treatment increases the expression of TET3 in the same cell type, but estradiol only induces the expression of TET1 in stromal cells, indicating that sex hormones regulate the expression of TET genes in a dynamic and cell-specific manner in the human endometrium (27). Our previous study found that the expression of TET3 gradually decreases in the endometrial tissues of women in the proliferative and secretory phases of the menstrual cycle as well as in the decidual tissues of early pregnancy, whereas it increases in the decidual tissues of women with EPL. Further mechanism studies indicate that TET3 negatively mediates miR-29a’s role in promoting the decidualization of endometrial stromal cells (ESCs) *in vitro* and maintaining pregnancy *in vivo*, suggesting that TET3 inhibits decidualization of ESCs, which may be involved in the pathogenesis of EPL caused by abnormal decidualization (35).

It is worth noting that miR-29a can upregulate the levels of decidualization markers IGFBP1 and PRL, whereas TET3 inhibits this effect (35). The specific mechanism remains to be further explored. At present, there are two main mechanisms of action of TET3: first, TET3 α-ketoglutaric acid (α-ketoglutarate, α-KG) and Fe2+catalyze the conversion of 5mC to 5hmC, mediate the demethylation process, and finally promote gene expression (36). In addition to the above mechanisms, TET3 also combines O-linked β-*N*-acetylglucosamine (*O*-GlcNAc) transferase (OGT), catalyses the *O*-GlcNAc glycosylation of histone serine and threonine residues (*O*-GlcNAcylation), and the final effect is to promote the downstream target genes transcription (37). However, these mechanisms are not enough to explain the phenomenon that TET3 downregulates the levels of IGFBP1 and PRL. There should be other mechanisms by which TET3 regulates the decidualization process of ESCs.

Based on the epigenetic modification mechanism, it can reduce or enhance the degree of DNA aggregation, thus regulating the expression of target genes at the transcriptional level (38–40). At the same time, other members of the TET family, TET1 and TET2, bearing certain structural homology with TET3, are also proved to be able to combine with multiple epigenetic regulatory molecules, for example, TET1 combines SIN3A, MeCP2, HDAC1/6/7, EZH2, LSD1, etc. (41, 42); TET2 combines Smarcb1/c2/e1, HDAC1/2, Ncor1/2, Baz1a/1b, Top2a/2b, Mbd2, Phf2, Ino80, Sap30bp, Trrap, Wdhd1, Chd8, Chaf1a, and Dnmt3a, etc. (43). At present, studies have shown that multiple molecules interacting with TETs play a role in the decidualization of ESCs, including SIN3A, EZH2, Dnmt3a, etc. (11, 44, 45), suggesting that members of the TET family can not only act as catalytic enzymes to affect epigenetic modification, but also act as anchor proteins for a variety of epigenetic modification enzymes.

Besides this, some studies also show TET expression in endometrial pathology. For example, a higher level of TET3 and lower levels of TET1 and TET2 were found in endometrial cancer compared with the normal endometrium, whereas endometrial cancer tissues showed lower levels of global hydroxymethylation at the same time (46). TET gene expression was also found dysregulated in the ectopic endometrial tissue of women with endometriosis, including decreased *TET1* levels (47). However, the expression and regulation of TETs in the endometrium is still not clear. Therefore, further studies are required to explore the mechanism by which and how TETs regulate the key processes during decidualization, embryo implantation, and placental growth.

## Additional biological roles of TET enzymes in decidual immune tolerance

### Decidual immune microenvironment and DNA methylation levels

Decidual immune cells are mainly composed of natural killer (NK) cells, macrophages, T cells, dendritic cells, and so on. Decidual NK (dNK) cells represent the largest population (50%–70%), whereas macrophages comprise approximately 10%–20% of whole decidual leukocyte populations, and the others are a very small minority (48–50). These immune cells, together with decidual stromal cells, cooperate to modulate trophoblast invasion, promote fetal growth, and regulate immune tolerance. Epigenetic modifications, including DNA methylation, are a key avenue for controlling immune responses, which can change the gene expression level without altering the underlying DNA sequence, thus allowing for a rapid adaptation of cells to the surrounding environment (51, 52). DNA methylation also provides an unexplored mechanism for immune regulation of decidual immune cells during the endometrial cycle, which could help explain how decidual immune cells are able to adapt and respond to the dynamic changes throughout the decidualization process. Interestingly, one recent study has identified low expression levels of genes that are related to NK cell function, such as KIR2DL3 and KLRC3, at the late proliferative phase, suggesting a decreased immune response mediated by NK cells at this phase of the endometrial cycle, which is consistent with the modulation of the immune response to favor embryo implantation (53). Besides this, another study also found the genes affected by decreased methylation were mainly associated with immune response regulation (FYN, BCL3, PVR, JAK3, IL1RL1, RFTN1, MYO1G, CXCL13, and C1S) (19).

### Roles of TET enzymes in immune cell development and function

Whereas the implication of TET proteins in DNA demethylation is well-established, the mechanisms underlying TET proteins in immune cells is yet to be explored. Strikingly, TET loss of function is strongly associated with hematological malignancies. For example, TET2 loss-of-function mutations are frequently observed in myelodysplastic syndromes and myeloid malignancies as well as in certain peripheral T-cell lymphomas (54–57). The biological roles of TET proteins in immune cell development, function, and malignant transformation have been unraveled in these studies.

### T cells

In T cells, the loss of TET proteins may result in compromised immune function or malignant transformation. Of note, TET2/3 are preferentially expressed in T cells compared with TET1 and play central roles in 5hmC modification in these cells (58). Deletion of TET2 alone in the hematopoietic system or in T cells did not result in any defect in T-cell lineage fate, indicating a compensative relationship between TET2 and TET3 (59, 60). However, it is reported that lack of TET2 enhanced CD8^+^ T-cell memory formation and differentiation (60). Although deletion of TET2 in CD4^+^ T cells in mice have intact thymic and peripheral T-cell subpopulations, typical cytokine expression was found decreased, including IL-17, IL-10, and IFN-g (59). The most profound phenotypes have been found in T cells upon codeletion of at least two TET members. For instance, TET2/TET3 DKO mice exhibited a striking increase of iNKT cells with impaired function and enhanced stemness (59, 61, 62). Surprisingly, genome-wide DNA methylation remains unchanged in response to the loss of TET proteins, but the deposition of 5hmC across specific genes, such as Tbx21 and Zbtb7b, is affected, suggesting a focal regulation role of TET members. These TET2/3 DKO iNKT cells can produce large amounts of immune response–related cytokines and drive other immune cell subset expansion and responses. In addition, TET enzymes are also required for the homeostasis of T regulatory (Treg) cells by modulating the expression of the transcriptional factor FOXP3. TET2 and TET3 are able to demethylate two intronic enhancers, termed conserved noncoding sequence (CNS) 2, which is critical for maintenance of FOXP3 expression (63–65). Deleting TET2 and TET3 specifically in Tregs not only results in compromised Treg lineage, but also a gain of aberrant activation and effector function in those cells, which enhances whole-body inflammation and ultimately accelerates death. Double TET1/2 deletion may also result in impaired Treg inactivation and differentiation due to hypermethylation of the CNS locus (66). Given the importance of all kinds of T cells involved in the endometrial cycle and decidualization process, future pharmacological methods specifically targeting TET proteins to modulate T-cell activity may employ a strong biological effect in the endometrial cycle.

### B cells

TET-dependent DNA demethylation is essential for B-cell differentiation, maturation, and function. TET protein expression levels are dynamically regulated during B-cell development. TET1 is significantly reduced in pro-B-cells, whereas TET2 and TET3 is increased during B-cell maturation and activation, suggesting a critical role of TET proteins in B-cell biology (67). *In vitro* analysis of TET1 KO cells showed a promoted status of lymphoid bias differentiation with more self-renewing pro-B-cell colonies compared with pre-B-cells (68). Long-term lack of TET1 resulted in lymphocytosis in mice by 18–24 months of age. TET2, one of the most frequently mutated genes in diffuse large B-cell lymphoma, works as a tumor-suppressor gene. Based on previous studies, TET2 was shown to be required for CSR and affinity maturation of antibodies, and disruption of TET2 may result in germinal hyperplasia. Mechanically, TET2 can preferentially strengthen the activity of enhancers (Igk and Aicda) (69). Compared with TET2 deletion, codeletion of TET2 and TET3 may cause more severe B-cell phenotypes during bone marrow development, including halting the pro-B-cell to pre-B-cell transition process and decreasing mature B cells in mice, and it diminishes the rearrangement of the Igk locus by increasing CpG methylation levels at the Igk3′ and distal enhancers (70). Future studies are needed to examine how TET proteins epigenetically affects B-cell biology in the decidual microenvironment.

### Myeloidcell

Compared with other TET proteins, TET2 is preferentially abundantly expressed in myeloid cells and further required for the myeloid cell–mediated innate immune response and surely critical in the decidual immune microenvironment (71, 72). TET2 deletion does not dramatically alter alternative macrophage (M2) gene expression levels, but indeed decreases the immunosuppressive function of these cells. TET2-KO macrophages and DCs produce more proinflammatory cytokines, such as IL-6, in response to bacterial activation (43, 73). Compared with wild-type mice, Tet2-KO mice show increased susceptibility to endotoxin-induced shock, DSS induced colitis, and so on, all suggesting the anti-inflammatory function of TET2 (43). Notably, during tumor growth, TET2 expression was found increased in myeloid-derived suppressor cells and tumor-associated macrophages and preserved immunosuppressive gene expression levels. TET2 deficiency in tumor-associated macrophages results in defective immunosuppressive capacity and an altered cytokine expression profile (74, 75). However, the role of TET2 in the myeloid-mediated decidualization process awaits further investigation.

### NK cells

NK cells play central roles in boosting inflammation and decidualization, but the evidence is lacking regarding whether and how TET proteins function in NK cells and, thus, have an effect during the endometrial cycle (3, 48). Continued efforts are needed to investigate the possible role of TET proteins in dNK cells, and this will shed light on the current understanding of the biological role of TET enzymes in decidual immune tolerance.

### Macrophages

Macrophages are highly diverse cells and the major antigen-presenting cells at the maternal-fetal interface. In addition to protection of the embryo from the attack of the maternal immune system, decidual macrophages also play a key role in embryo implantation, trophoblastic invasion, spiral artery remodeling, and placentation. Recently, new concepts have emerged to explain how macrophage polarization and function are regulated, including immune metabolism and epigenetics (76, 77). Macrophages are divided into M1-like macrophages and M2-like macrophages. M1 macrophages secrete a variety of cytokines including IL-2, IL-6 and TNF-α, which involved in pro-inflammatory responses, whereas M2 macrophages are mainly involved in anti-inflammatory responses (78). The balance of M1 macrophages and M2 macrophages is critical for various processes in both normal and pathological pregnancy (79). However, the functions of TETs in decidual macrophages are largely unknown. Although only a few studies showed epigenetic regulation in the differentiation and function of decidual macrophages, emerging studies reported the role of epigenetic modulating by TETs in macrophages in other fields (43, 73, 80), which may shed new insights for further studies on decidual macrophages.

## Conclusions and future perspectives

Collectively, TET proteins are critical to 5hmC/5Mc/5fC/5caC modification in various decidual immune and stromal cells, which is essential for the decidualization process and early pregnancy. It is expected that using specific compounds modulating TET activity may be useful in pregnancy-related diseases and for modulating immune cell responses during the decidualization process. Thus, it is critical to elucidate the functional role of TET proteins for modulating 5hmC/5mC/5fC/5caC levels in decidual stromal and immune cells, which requires further understanding of the possible underlying molecular mechanism in various cell types. Future work may also be required to explore how to discover and utilize novel TET interactors to modulate immune responses during decidualization and early pregnancy. The elucidation of these aspects will open an exciting field for future work.

## Author contributions

MJ conducted the literature search and completed the manuscript of the first draft in collaboration with JJ. XC and YZ helped prepare the manuscript. DW and AL revised and edited the final version of the manuscript. All authors contributed to the article and approved the submitted version.
